# Modulating glioblastoma chemotherapy response: Evaluating long non-coding RNA effects on DNA damage response, glioma stem cell function, and hypoxic processes

**DOI:** 10.1093/noajnl/vdac119

**Published:** 2022-08-10

**Authors:** Edith Yuan, Kristie Liu, Justin Lee, Kathleen Tsung, Frances Chow, Frank J Attenello

**Affiliations:** Keck School of Medicine, University of Southern California, Los Angeles, CA, USA; Keck School of Medicine, University of Southern California, Los Angeles, CA, USA; Department of Neurological Surgery, Keck School of Medicine, University of Southern California, Los Angeles, CA, USA; Department of Neurological Surgery, Keck School of Medicine, University of Southern California, Los Angeles, CA, USA; Department of Neurological Surgery, Keck School of Medicine, University of Southern California, Los Angeles, CA, USA; Department of Neurological Surgery, Keck School of Medicine, University of Southern California, Los Angeles, CA, USA

**Keywords:** chemo-response, glioblastoma, long non-coding RNAs, Temozolomide

## Abstract

Glioblastoma (GBM) is the most common and aggressive primary adult brain tumor, with an estimated annual incidence of 17 000 new cases in the United States. Current treatments for GBM include chemotherapy, surgical resection, radiation therapy, and antiangiogenic therapy. However, despite the various therapeutic options, the 5-year survival rate remains at a dismal 5%. Temozolomide (TMZ) is the first-line chemotherapy drug for GBM; however, poor TMZ response is one of the main contributors to the dismal prognosis. Long non-coding RNAs (lncRNAs) are nonprotein coding transcripts greater than 200 nucleotides that have been implicated to mediate various GBM pathologies, including chemoresistance. In this review, we aim to frame the TMZ response in GBM via exploration of the lncRNAs mediating three major mechanisms of TMZ resistance: (1) regulation of the DNA damage response, (2) maintenance of glioma stem cell identity, and (3) exploitation of hypoxia-associated responses.

Glioblastoma (GBM) is the most common primary and aggressive adult brain tumor, with an estimated annual incidence of 17 000 new cases in the United States.^1^ Therapies targeting different pathological mechanisms, such as aberrant signaling pathways and angiogenesis, have been developed over the years. Despite these efforts, limited effective therapeutic options are available. The 5-year survival rate remains at a dismal 5%, with a median survival period of 14.6 months. Currently, the standard treatment entails maximal surgical resection followed by chemotherapy and radiation.^2^

Temozolomide (TMZ) is a first-line chemotherapy drug for GBM. TMZ exerts an antitumor effect via methylation of DNA at the *O*^6^ position of guanine, forming *O*^6^-methylguanine that incorrectly pairs with thymine. This base mispairing induces continuous activation of the mismatch repair (MMR) pathway and DNA double strand breaks (DSBs), which eventually results in cell cycle arrest and apoptosis (Figure 1A).^3^ Concomitant treatment with TMZ and radiation for GBM has been shown to increase the median survival rate from 12.1 to 14.6 months when compared to radiation alone.^4^

**Figure 1.**
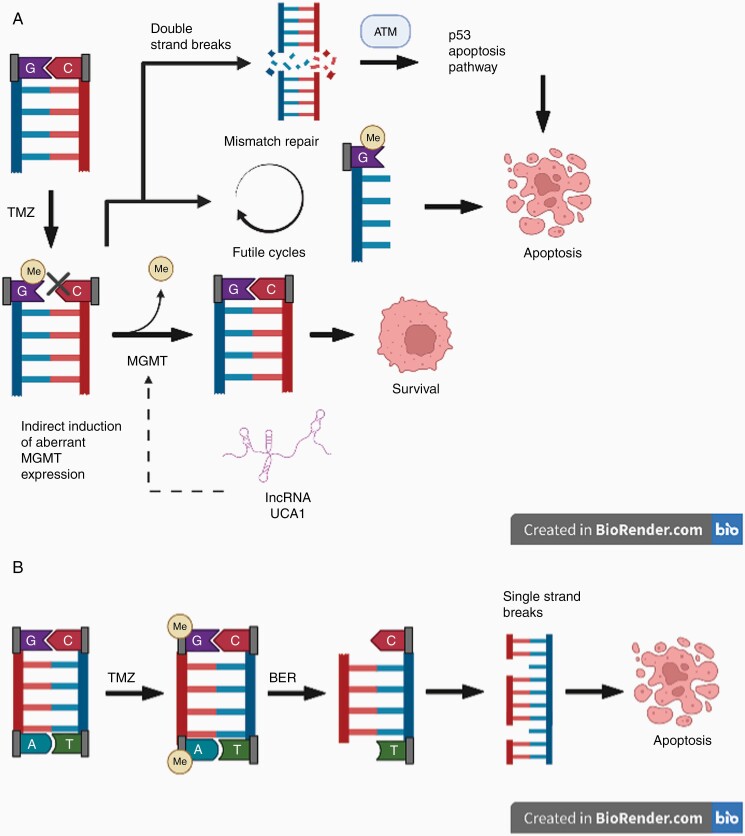
(A) Schematic diagram of TMZ and its relationship to the MMR, DSB, and MGMT pathways. TMZ adds a methyl group to guanine at the *O*^6^ position, creating *O*^6^-methylguanine that incorrectly pairs with thymine. The mispairing continuously activates the MMR pathway, resulting in DSBs and apoptosis. MGMT removes the methyl group from *O*^6^-methylguanine and prevents the activation of the MMR pathway. LncRNA *UCA1* is shown to indirectly increase MGMT expression level. (B) Schematic diagram of TMZ and the BER and SSB pathways. TMZ adds a methyl group to guanine and adenine, forming *N*^7^-methylguanine and *N*^3^-methyladenine that are recognized by the BER pathway. When the bases are excised, SSBs result, creating instability that results in cell death. Me, methyl; G, guanine; C, cytosine; T, thymine.

Despite the survival advantage of TMZ, a large percentage of GBMs exhibits resistance to the chemotherapy. TMZ resistance may result from either intrinsic or acquired mutations and is thought to represent one of the main causes of treatment failure and tumor recurrence. Studies have previously focused on well-recognized contributors to TMZ resistance including *O*^6^-methylguanine-DNA methyltransferase (MGMT) DNA damage response (DDR), MMR defects, as well as presence of cancer stem cells, and tumor hypoxia.^5–8^ However, it has been increasingly recognized that long non-coding RNAs (lncRNAs) play an extensive role in mediating various aspects of GBM pathology, including the chemoresistance mechanisms described here.

LncRNAs are nonprotein coding transcripts longer than 200 nucleotides that have emerged as critical regulators of gene expression under normal physiology as well as disease states.^9^ Studies have implicated lncRNAs in contributing to GBM pathogenesis by regulating gene expression at the epigenetic, transcriptional, and posttranscriptional levels.^10^ For instance, *TUG1* is one of many lncRNAs that interacts with chromatin-modifying proteins responsible for histone methylations of genes critical for GBM progression.^11^ At the posttranscriptional level, many lncRNAs behave as competing endogenous RNAs that act as molecular sponges for micro-RNAs (miRNAs). These interactions disinhibit the miRNA’s downstream pathways and proteins, such as the Notch pathway and the VEGFA axis that promote glioma cell proliferation and angiogenesis, respectively.^12,13^

In the past few years, studies have explored the association between lncRNAs and TMZ response in GBM. The rising number of lncRNAs discovered may offer novel insights into GBM resistance to TMZ and represent potential therapeutic targets. Notably, while other reviews have discussed chemo-response in the context of lncRNAs, most prior reviews have focused on collections of well-known lncRNAs associated with chemo-sensitivity. Furthermore, prior reviews have also primarily concentrated on common lncRNAs noted to have chemoresistance roles without extensive exploration of chemosensitivity of specific lncRNAs.^14^ In contrast, this review aims to frame the TMZ response in GBM via exploration of the lncRNAs mediating the three major mechanisms of TMZ resistance: (1) regulation of the DDR, (2) maintenance of glioma stem cell (GSC) identity, and (3) exploitation of hypoxia-associated responses (Table 1).

**Table 1.** Summary of Mechanisms on LncRNA and TMZ Response

Mechanism	LncRNA	Expression Level in TMZ Resistant GBM	Sample Used	Summary of Mechanism on TMZ Response	ceRNA	References
I. DNA damage response						
MGMT						
	*Lnc-TALC*	Upregulated	Patient samples and LN229, U251	Increases MGMT expression through regulating c-met and stat3	*miR-20b-3p*	^15^
	*FOXD2-AS1*	Upregulated	U251 and A172	Increases MGMT expression	N/a	^16^
		Upregulated	U87, U251, LN229, A172	IncreasesCPEB4 (unrelated to MGMT)	*miR-98-5p*	^17^
	*UCA1*	Upregulated	Patient samples and A172, T98G, SHG44	Increases MGMT Expression	*miR-182-5p*	^18^
MMR	*XIST*	Upregulated	Patient samples and U251, U373, LN229, U118, LN229	Increases MSH6 indirectly through SP1, leading to abnormal MMR activity	*miR-29c*	^19^
DSB	*SBF2-AS1*	Upregulated	U87, LN229, A172, T98, U251	Enhances DSB repair by increasing XRCC4	*miR-151a-3p*	^20^
II. Glioma stem cells	*TP73-AS1*	Upregulated	G26 and G7 glioma stem cell lines	Highly upregulated in GSC and may mediate its effect through regulating *ALDH1A1*	N/A	^21^
	*SOX2OT*	Upregulated	Patient samples and U87, U251	Knockdown decreases SOX2 expression through methylating its promoter	N/A	^22^
	*NEAT1*	Upregulated	Patient samples and U87, U251	Highly upregulated in GSCs and activates MAP3K1	*let7g-5p*	^23^
		Upregulated	Patient samples	Activated by HMGB1 as part of the Wnt Pathway		^24^
	*Linc00174*	Upregulated	Patient samples and LN229, SHG44, U118, U251, U87	Maintains SOX9 expressions to enhance GSC-like behavior	*miR-138-5p*	^25^
	*BC200*	Upregulated	Patient samples, T98G, U87	Overexpression promoted GSC behaviors and TMZ resistance	*miR218-5p*	^26^
III. Hypoxia						
EMT	*MALAT1*	Upregulated	U251, U87	Knockdown decreases ZEB1	N/A	^27^
		Upregulated	U251	Increases GSK3ß, MGMT (unrelated to EMT)	*miR-101*	^28^
		Upregulated	Patient samples, U87, U251	Increases thymidylate synthase (unrelated to EMT)	*miR-203*	^29^
	*RP11-838N2.4*	Downregulated	Patient samples and U87, U251	Knockdown is associated with downregulation of EphA8	*miR-10a*	^30^
	*H19*	Upregulated	U251, M059J	Stimulates the Wnt/beta-catenin pathway	N/A	^31^
			U251, LN229	Stimulates the NF-kB pathway	N/A	^32^
Metabolism	*HOTAIR*	Upregulated	Patient samples and U87, A172	Knockdown suppresses HK2 and increases TMZ-induced apoptosis	*miR-125*	^33^
			Patient samples and serum	Increases EVA1 expression indirectly by inhibiting *miR-256b-3p*	*miR-526b-3p*	^34^
IV. Miscellaneous	*AC003092.1*	Downregulated	Patient sample and U87	Overexpression increases TFPI-2 and TMZ-induced apoptosis	*miR-195*	^35^
	*CASC2*	Downregulated	Patient samples, U251, U373, SNB19, U118, LN229	Knockdown leads to decreased PTEN and diminished TMZ response	*miR-181a*	^36^
	*TUSC7*	Downregulated	U87	Overexpression suppresses expression of multidrug protein 1	*miR-10a*	^37^
	*ADAMTS9-AS2*	Upregulated	Patient samples, T98G, U118	Overexpression upregulates the FUS/MDM2 Ubiquitination axis	N/A	^38^

Abbreviations: CPEB4, cytoplasmic polyadenylation element binding protein 4; GSK3ß, glycogen synthase kinase 3 Beta; EVA1, epithelial v-like antigen 1; TFPI-2, tissue factor pathway inhibitor-2; *CASC2*, cancer susceptibility candidate 2; PTEN, phosphatase and tensin homologs; *TUSC 7*, tumor suppressor candidate 7; FUS, fused in sarcoma; MDM2, murine double minute 2.

## LncRNAs and the DDR

The DDR is a network of cellular processes that identifies and repairs DNA damage to prevent mutations and other genetic aberrations that threaten cell viability. Direct DNA-lesion reversal, MMR, base excision repair (BER), DNA DSB, and single-strand breaks (SSBs) repair are all components of the DDR. Dysregulated DDR pathways lead to the accumulation of DNA damage, evasion of cell cycle arrest, and chemoresistance. DSBs have an important role in mediating TMZ-induced apoptosis by triggering DNA damage kinase, ATM serine/threonine kinase, which then activates the p53-driven apoptosis pathway.^39^ In addition to *O*^6^-methylguanine, TMZ also forms *N*^7^-methylguanine and *N*^3^-methyladenine that are recognized and repaired by the BER pathway, specifically by *N*-methylpurine DNA glycosylase and DNA polymerase β. The abasic sites generated from the DNA glycosylase in the BER pathway are inherently unstable and readily convert into DNA SSBs; unrepaired SSBs can collapse the DNA replication pathway resulting in cell death (Figure 1B).^40^

LncRNAs have been identified to utilize the DDR pathways as a method of modulating treatment resistance. *SBF2-AS1*, which is upregulated in TMZ-resistant GBM cells, functions as a ceRNA against *miR-151a-3p* to enhance X-ray repair cross-complementing 4 (XRCC4) protein levels, augmenting DSB repair in gliomas.^20^ Radiation-induced DNA damage can similarly activate lncRNAs. *HMMR-AS1* was found to be elevated after irradiation along with increased expression of DDR proteins ATM, RAD51, and BMI1, ultimately promoting glioma cell growth.^41^ Below, we will describe in further detail on how lncRNAs regulate TMZ resistance through direct DNA-lesion reversal and MMR.

### LncRNA and MGMT

MGMT is a DNA methyltransferase that augments TMZ resistance by removing TMZ-induced alkyl groups and preventing the mispairing between *O*^6^-methylguanine and thymine (Figure 1B). As part of the direct DNA-lesion reversal process, MGMT expression is induced with alkylating agents and serves as an important prognostic marker.^42^ Clinical trials have demonstrated that MGMT promoter CpG methylation is associated with significantly improved overall survival in primary and recurrent GBM patients.^5,43^

However, even though clinical data on direct MGMT inactivators, such as *O*^6^-benzylguanine and *O*^6^-(4-bromothenyl) guanine (*O*^6^-BG), showed improved survival in patients simultaneously treated with TMZ, TMZ was associated with increased hematopoietic toxicity due to nonspecific targeting, indicating that additional, more precise therapies targeting MGMT are required.^44^ Because lncRNAs are highly cell type-specific, targeting lncRNAs as a method of regulating MGMT expressions may represent promising alternatives.^10^ Here, we summarize the data on lncRNA-mediated epigenetic regulation of MGMT and the effect on TMZ resistance in GBM.

#### Lnc-TALC.

—TMZ-associated lncRNA in GBM recurrence (*lnc-TALC*) is a newly identified lncRNA highly expressed in patient-derived TMZ-resistant GBM tissues. Clinically, GBM patients with lower *lnc-TALC* expression exhibit a significantly better response to TMZ with a lower likelihood of tumor recurrence.^15^ To determine the association between *lnc-TAC* and TMZ-resistance, Wu et al. conducted clustered regularly interspaced short palindromic repeats (CRISPR)-induced *lnc-TAC* knockdown in TMZ-resistant human GBM cell lines, LN229R and U251R, and patient-derived GBM cells. The cells with decreased *lnc-TALC* had reduced MGMT mRNA and protein levels and increased TMZ-induced apoptosis. LN229R cells with *lnc-TALC* knockdown were then implanted in TMZ-treated mice; these mice showed significantly longer survival and smaller tumors when compared to mice with LN229R xenografts. This suggested that decreased *lnc-TALC* was associated with restored TMZ sensitivity in GBM cells. Additional in vitro experiments were conducted to delineate the mechanism linking higher *lnc-TALC* expression with TMZ resistance. A luciferase bioluminescent reporter assay confirmed that *lnc-TALC* functions as a ceRNA against *miR-20b-3p* to indirectly promote MGMT expression. Through sequestration of *miR-20b-3p*, *lnc-TALC* upregulates c-MET and its downstream target Stat3. Protein coimmunoprecipitation analysis subsequently demonstrated that Stat3 binds to a histone acetyltransferase to increase the acetyltransferase affinity to the *MGMT* promoter, effectively increasing MGMT expression.^15^

#### FOXD2-AS1.

—FoxD2 adjacent opposite strand RNA 1 (*FOXD2-AS1*) is a lncRNA highly expressed in recurrent GBM tumors and is associated with decreased patient survival.^45^ A study showed that suppression of *FOXD2-AS1* via a small interfering RNA (siRNA) is associated with decreased GBM cell viability in TMZ.^16^ While the specific mechanism is unclear, the study reported that *FOXD2-AS1* may oppose TMZ cytotoxicity through MGMT upregulation. siRNA-induced suppression of *FOXD2-AS1* (si-*FOXD2-AS1*) in human GBM cell lines, U251 and A172, is associated with hypermethylation of the MGMT promoter, resulting in decreased MGMT mRNA and protein levels. Furthermore, the si-*FOXD2-AS1* GBM cells showed decreased proliferation and increased cell death when treated with TMZ. This short and straightforward study indicated that *FOXD2-AS1* downregulation promotes TMZ sensitivity in GBM cell lines by decreasing MGMT expression.^16^

*FOXD2-AS1* also drives TMZ resistance as part of the *FOXD2-AS1/miR-98-5p/*CPEB4 axis unrelated to MGMT regulation. A separate study found that *FOXD2-AS1* knockdown inhibited cell proliferation and promoted TMZ-induced apoptosis. RNA pull-down assay indicated that *FOXD2-AS1* serves as a ceRNA to adsorb *miR-98-5p*. Computational analysis showed that *miR-98-5p* and cytoplasmic polyadenylation element binding (CPEB4) shared binding sites. An oncogenic RNA binding protein, CPEB4 is associated with glioma migration, growth, and vascularization. The current study showed that expression of CPEB4 was inhibited by knockdown of *FOXD2-AS1* or upregulation of *miR-98-5p*, leading to decreased apoptosis and increased survival in vitro.^17^

#### UCA1.

—A recently published paper by Cheng et al. demonstrated that the urothelial cancer-associated 1 (*UCA1*) lncRNA modulates TMZ response through MGMT. Identified as an oncogene, *UCA1* expression was directly correlated to glioma grade and MGMT expression. Transfection of GBM cells, A172 and SHG44, with si-*UCA1* showed decreased glioma cell viability with increased cell apoptosis. The si-*UCA1* cells also exhibited decreased MGMT protein level. *In vivo* experiments showed that the tumors with smallest volume and weight were from TMZ-treated mice implanted with si-*UCA1* GBM cells. The authors also identified *miR-182-5p* as a target of *UCA1*, forming the *UCA1/miR-182-5p*/MGMT axis that modulates MGMT expression and TMZ sensitivity.^18^

### LncRNA and MMR

MMR is an important postreplication mechanism that recognizes and repairs nucleotide mismatches. In TMZ-treated cells, MMR is activated to excise the mismatched thymine. Because *O*^6^-methylguanine remains on the template strand, this leads to futile cycles of thymine mispairing and excising that eventually results in DNA DSBs and cell death.^46^

Deficiencies in MMR render tumors resistant to TMZ, regardless of the MGMT methylation status.^7^ Aberrations in MMR proteins, such as melanocyte-stimulating hormone 2, MSH6, and mutL homolog 1, or defective DSB repairs can lead to suppressed apoptotic response and increased survival.^46–49^ Furthermore, while MSH6 mutations were not observed in pre-TMZ treated GBM tissues, 26% of recurrent tumors post-TMZ treatment had decreased MSH6 expression, suggesting that loss of MSH6 function is associated with tumor recurrence during TMZ treatment.^46^ Currently, the relationship between lncRNAs and MMR is considerably understudied and deserves additional extensive investigations. Thus far, only the lncRNA X-inactive specific transcripts (*XIST*) has been identified to alter the TMZ response in GBM through modulating the MMR.

#### XIST.

—*XIST* is a well-characterized lncRNA in various cancers, including GBM.^50^ Paired analysis between patient-derived GBM and peritumoral brain tissues has revealed *XIST* to be significantly higher in the former. More importantly, *XIST* is directly associated with larger tumor size and overall shorter survival period.^50^ Human GBM cells transfected with a siRNA targeting *XIST* displayed diminished proliferation rate and increased sensitivity to TMZ as demonstrated by the BrdU and viability assay, respectively.^51^ RNA immunoprecipitation further showed that *XIST* affects the TMZ response by directly binding *miR-29c* and inhibiting its expression. This interaction results in upregulation of specificity protein 1 (SP1), a transcription factor that causes aberrant expression of the MMR-associated protein, MSH6.^19^ Taken together, the result suggests GBM cells with elevated *XIST* may exhibit decreased TMZ sensitivity due to the abnormal MSH6 expression.

## LncRNAs and GSCs

**Figure 2.**
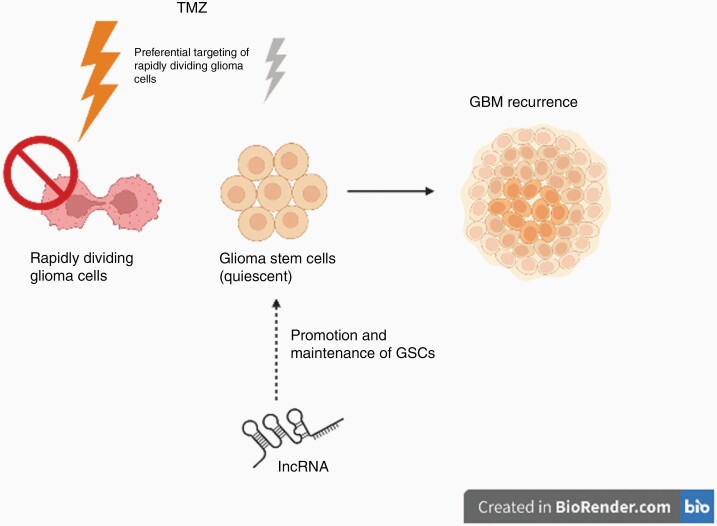
Diagram of the role of lncRNA in TMZ resistance through GSCs. As TMZ preferentially targets rapidly dividing glioma cells, the relatively quiescent glioma stem cells are more resistant to TMZ and are associated with GBM recurrence. LncRNAs, including *TP73-AS1* and *SOX2OT*, have been found to have a role in the promotion and maintenance of GSCs.

GBM harbors a subset of self-renewing population of cells termed GSCs that have been hypothesized to play a key role in GBM pathogenesis.^6^ As TMZ preferentially targets proliferating glioma cells, the relatively quiescent GSCs are favored to survive and increase the likelihood of GBM recurrence. Notably, xenograft mouse models of human GBM cells have demonstrated that post-TMZ administration, the majority of residual, treatment-resistant tumor cells were fluorescently labeled GSCs with the ability to reinitiate GBM.^6^ In addition, CD133^+^ GSCs exhibit higher levels of MGMT mRNA and lower expressions of autophagy- and apoptotic-related proteins that allow them to evade conventional therapies (Figure 2).^52–54^

Previous literature has illustrated that irregular expression of specific lncRNAs are closely related to the malignant phenotypes of GSCs. For instance, lncRNAs *XIST*, *H19,* and *MIAT* interact with transcription factors that promote and maintain GSCs in vitro.^55^ High throughput microarray profiling studies in GSCs have further identified differential expressions of specific lncRNAs to be associated with GSC behaviors, such as neurosphere formation and therapy resistance.^56,57^ GSC-associated lncRNAs represent an innovative strategy for targeting GSCs. One study has identified 1545 unique lncRNAs found to regulate GSCs’ differentiation status; the lncRNAs identified are highly specific, with the ability to promote differentiation in GSCs and decrease the tumor cells’ tumorigenicity.^58^ Studies described below have investigated how different lncRNAs affect the TMZ response through regulating GSC-like behaviors.

### TP73-AS1

In GBM, lncRNA P73 antisense RNA 1T (*TP73-AS1*) expression level is elevated in patient-derived GSCs and correlates directly with early mortality.^21^ Mazor et al. used CRISPR to downregulate *TP73-AS1* in two established human GSC lines and noted that the cells exhibited decreased viability under TMZ treatment. Further elucidating the mechanism underlying *TP73-AS1-*associated TMZ resistance, transcriptome analysis of the *TP73-AS1*-attenuated GSCs revealed perturbations of neuronal differentiation and metabolic reprogramming pathways. One of the transcripts found to be particularly downregulated was aldehyde dehydrogenase 1 family member A1 (*ALDH1A1).*^21^ ALDH1A1 is recognized as a marker of GSCs and a promoter of clonogenicity; its expression is also elevated in recurrent, TMZ-resistant GBMs.^59^ Moreover, treating GSCs with a pharmacological ALDH1A1 inhibitor significantly increased their sensitivity to TMZ, suggesting that *TP73-AS1* may mediate its effects on TMZ resistance through regulating *ALDH1A1*.^21^

### SOX2OT

LncRNA SOX2 overlapping transcript (*SOX2OT)* is mapped to the human chromosome 3q26.3 and harbors the SRY-Box Transcription Factor 2 (*SOX2)* gene in its intronic region.^60^ Analysis of patient-derived GBM samples has shown *SOX2OT* level to positively correlate with both advanced glioma grade and reduced median survival time.^61^ In vitro studies showed that GBM cells U87 and U251 transfected with *SOX2OT* siRNA lentiviral vectors (si-*SOX2OT)* displayed increased TMZ-mediated apoptosis. As lncRNAs can exert cellular functions through regulating proximal genes, the authors predicted *SOX2OT* modulates the TMZ response in GBM through upregulating SOX2. This was confirmed in si-*SOX2OT* U87 and U251 cells demonstrating significantly decreased SOX2 mRNA and protein levels.^22^ By de-differentiating tumor cells to GSCs, SOX2 is one of the four core transcription factors essential for GBM propagation.^62^ Downregulation of SOX2 interferes with GSCs’ ability to self-renew and consequently, to form gliomas.^63,64^ Importantly, SOX2 has previously been established to take part in mediating TMZ resistance in GBM.^65^ Mechanistically, *SOX2OT* maintains SOX2 expression by interacting directly with α-ketoglutarate-dependent dioxygenase alkB homolog 5, an RNA demethylase that binds to the SOX2 promoter and is similarly upregulated in TMZ-resistant GBMs.^22^

### NEAT1

The lncRNA nuclear paraspeckle assembly transcript 1 (*NEAT1*) is overexpressed in GBM tissues and is a marker of poor survival in primary GBM patients.^66^*NEAT1* is upregulated in GSCs and plays an important role in augmenting their proliferation rate and resistance to chemotherapy. Induced by high-mobility group box 1 in GBM cells treated with TMZ, *NEAT1* has been shown to promote GSC formation through the Wnt/β-catenin pathway.^24^ In addition, a recent study showed that GSCs transfected with short hairpin RNA (shRNA) against *NEAT1* (sh-*NEAT1*) are associated with significantly reduced viability and proliferation when exposed to TMZ in vitro. Using a bioluminescence assay, the authors identified *let7g-5p* as a downstream target of *NEAT1*. A prior study had demonstrated *let7g-5p* as a tumor-suppressive miRNA associated with impaired GSC behavior.^67^ Bioinformatic analysis predicted that *let7g-5p* shares a binding site with mitogen-activated protein kinase 1 (MAP3K1), a protein previously shown to correlate directly with shortened patient survival and increased TMZ resistance in GBM cells.^68^ Subsequent mRNA results showed that MAP3K1 expression was significantly increased in GBM cells transfected with *let7g-5p* shRNA. Through transcriptional and protein analyses, the authors concluded that *NEAT1* suppresses *let7g-5p* to activate MAP3K1 and ultimately inhibit the cytotoxic effects of TMZ.^23^

### LINC00174

As a relatively newly discovered lncRNA, *linc00174* is markedly upregulated in human GBM tissues and its expression level is inversely correlated with patient survival rate.^25^*Linc00174* has been reported to strengthen TMZ resistance in GBM cells by directly targeting *miR-138-5p*, a tumor suppressive miRNA shown to arrest GBM cell cycle.^69^ By inhibiting *miR-138-5p*, *linc00174* increases SRY-Box Transcription Factor 9 (SOX9), a downstream target of *miR-138-5p*. In gliomas, SOX9 has been shown to maintain the self-renewal ability of GSCs.^70^ GBM cells transfected with shRNA-mediated *linc00174* knockdown are associated with increased *miR-138-5p* and decreased SOX9 levels. In vitro, the transfected cells showed enriched apoptotic markers in addition to reduced viability and colony formation under TMZ exposure. The results indicated that inhibiting *linc00174* improves TMZ-induced apoptosis by discouraging stem cell-like behaviors through regulating *miR-138-5p* and SOX9.^25^

### BC200

In GBM tissues, Brain Cytoplasmic 200 (*BC200*) expression is significantly higher than in normal tissues. In addition, *BC200* was positively correlated with p53-mutation, indicating *BC200* association with poor prognosis in patients. Knockdown of *BC200* via siRNA suppressed common stem cell markers, including SOX2, and the self-renewal capacity of GBM cells. In contrast, GBM cells transfected with *BC200* overexpression vector demonstrated increased cell wound healing migration, invasiveness, and colony-forming ability. The cells also exhibited decreased TMZ resistance via viability assay. Western blot analysis showed increased MGMT, multidrug resistance protein, and ABC transporter. To further assess the effect of *BC200* on miRNA expression, bioinformatic analysis, and online database identified *miR-218-5p* level to be negatively associated with *BC200.* GBM cells with *miR-218-5p* inhibited showed higher TMZ resistance, stem cell markers, and colony-forming abilities. *In vivo* studies supported the *in vitro* experiments, demonstrating TMZ-treated mice implanted with si*BC200* GBM cells had significantly reduced tumor size and prolonged overall survival.^26^

## LncRNAs and Hypoxic Processes

**Figure 3.**
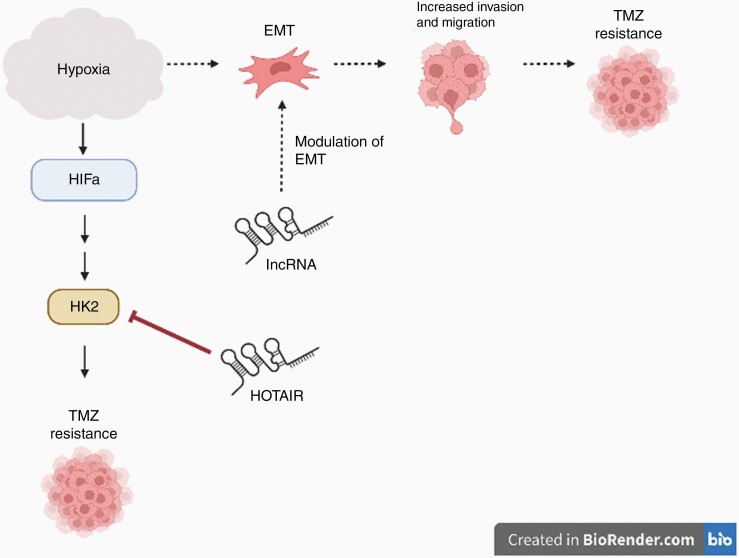
Diagram of the role of HOTAIR and other lncRNAs in TMZ resistance and hypoxia. Hypoxia encourages TMZ resistance through induction of EMT and activation of the HIFa pathway. *H19* and *MALAT1* have been shown to modulate the EMT, while HOTAIR is shown here to inhibit HK2 activity in the HIF-1α pathway.

The brain tumor microenvironment is composed of a heterogenous population of cancerous as well as nonneoplastic cells, including the surrounding tissue stroma and associated vascular supply. Tumor stromal cells secrete various angiogenesis-promoting factors where vascular endothelial growth factor is the most potent and its high expression level indicates poor outcome.^71^ As a result of poorly functional blood vessels, tumors exhibit extensive hypoxia and limited response to systemic chemotherapeutics. The presence of hypoxia upregulates gene expressions that promote cell survival and chemoresistance.^72^ Furthermore, the hypoxic niche hosts GSCs that stimulate endothelial cell growth and proliferation associated with neo-angiogenesis (Figure 3).^73^

Composed of several subunits (ie, HIF-1α, HIF-2α), hypoxia-inducible factor (HIF) is the main transcriptional regulator of cellular response to hypoxia. HIF overexpression promotes cancer angiogenesis, drug resistance, and certain lncRNAs.^8^ LncRNA *AWPPH* level correlates directly with HIF-1α and is associated with metastatic GBM patients. Silencing HIF-1α attenuated the enhanced glioma invasion and migration seen with elevated *AWPPH*.^74^ Consequently, the hypoxic microenvironment mediates two important processes implicated in the GBM TMZ resistance, the epithelial to mesenchymal transition (EMT) and metabolic alterations.

### LncRNA and EMT

The EMT is a process that transforms tumor cells into a more mesenchymal phenotype with enhanced migratory and invasive properties. The hypoxic environment promotes EMT through triggering the HIF1α/ZEB1 signaling axis.^75^ Cells undergoing EMT display decreased expression level of epithelial genes (ie, E-cadherin, ZO-1) and increased expression of mesenchymal genes (ie, N-cadherin and vimentin). These alterations lead to changes in cell morphology and promote stem cell-like features.^76^ Though EMT is most often associated with GBM invasion, it has been increasingly recognized for its contribution to chemoresistance.

Several studies have shown that the relationship between specific EMT proteins and lncRNAs regulates the chemotherapy response in different cancers. For example, in colorectal cancer, lncRNA *SLC25A25-AS1* increases the chemo-response through regulating EMT-associated proteins.^77^ Similarly, *lncRNA-ATB* inhibits the EMT proteins, ZEB1 and ZEB2, to increase trastuzumab resistance in breast cancer cells.^78^ Although the association between EMT and chemo-response necessitates more extensive investigation, understanding their relation to specific lncRNAs provides additional therapeutic avenues for patients with GBM.

#### MALAT1.

—In GBM, lncRNA metastasis-associated lung adenocarcinoma transcript 1 (*MALAT1*) is associated with poor TMZ response and survival in GBM patients. *MALAT1* is a well-studied lncRNA known to be upregulated following TMZ-induced alkylation damage and recognized to increase chemoresistance through various mechanisms, including upregulating GSK3ß, MGMT, and thymidylate synthase.^28,29,79^ A recent study described the direct association between *MALAT1*-mediated TMZ resistance and EMT. The investigators first transfected U87 and U251 GBM cells with *MALAT1* siRNA (si-*MALAT1*) to generate *MALAT1-*knockdown cells. After being treated with TMZ, the si-*MALAT1* cells showed reduced viability compared to the non-transfected cells. In addition, downregulation of ZEB1 was confirmed in the si-*MALAT1* cells at the mRNA and protein levels. Furthermore, *MALAT1*-associated TMZ resistance was reversed in U87 and U251 with siRNA-induced ZEB1 knockdown, suggesting that *MALAT1* enhances TMZ resistance via regulation of ZEB1.^27^ This data complements other studies that have indicated ZEB1 to facilitate TMZ resistance in GBM cells by elevating MGMT expression and accelerating double-stranded break repairs.^80^ Lastly, in mice implanted with patient-derived GBM cells, concurrent treatment of TMZ and a nanocomplex delivering si-*MALAT1* has been shown to significantly prolong survival, solidifying *MALAT1* as a lncRNA with promising therapeutic potential.^79^

#### RP11-838N2.4.

—*RP11-838N2.4* is a largely understudied lncRNA recognized for modulating the TMZ response in GBM. Reports of *RP11-838N2.4* and primary GBM tissues have revealed lower expression of *RP11-838N2.4* is associated with an increased risk of tumor recurrence and early mortality.^30^ Contrasting most other lncRNAs mentioned previously, *RP11-838N2.4* is inversely correlated with TMZ resistance. This is demonstrated when transfection of TMZ-resistant U87 (U87R) and U251 (U251R) GBM cells with a *RP11-838N2.4-*overexpressed plasmid exhibited increased TMZ-mediated cell death. Furthermore, the authors determined that *RP11-838N2.4* level is inversely associated with *miR-10a*, which negatively regulates its downstream target, ephrine tyrosine kinase receptor A8 (EphA8).^30^ Prior data has shown enhanced EphA8 reverses EMT and transforms glioma cells to the more cobble-stoned appearance of epithelial cells. In contrast, loss of EphA8 is associated with shorter survival time, and diminished TMZ response in GBM patients.^81^ Further mRNA and protein analysis indicated that *RP11-838N2.4* reduces the expression of *miR-10a* and relieves its inhibitory effect on EphA8, resulting in enhanced TMZ cytotoxicity by decreasing the EMT process.

#### H19.

—LncRNA *H19* is upregulated in human TMZ-resistant GBM tissues and is directly associated with decreased patient survival rate. Reports have shown *H19* to support TMZ resistance in GBM through different mechanisms, including catalyzation of EMT-related pathways.^31,32,82^ Studies have delineated the relationship between *H19*-associated TMZ resistance and the Wnt/β-catenin and NF-κβ processes, two signaling cascades known to induce both TMZ inefficiency and EMT.^31,32,83^ TMZ-resistant GBM cells transfected with *H19* shRNA had significantly lower half maximal inhibitory concentration (IC_50_) value for TMZ when compared to the control group. The *H19-*silenced cells also exhibited weaker signals of EMT-related and Wnt/β-catenin proteins. However, treatment with the Wnt/β-catenin activator, Licl, reversed the protein expressions and restored TMZ resistance in the *H19*-silenced cells.^31^ Similarly, *H19* has also been implicated to promote TMZ resistance in GBM through the NF-κβ pathway. U251 and LN229 GBM cells cotransfected with *H19* siRNA (si-*H19*) and a NF-κβ luciferase reporter showed decreased bioluminescence and mRNA levels of NF-κβ downstream targets, suggesting an association between *H19* and the NF-κβ pathway proteins. The si-*H19* cells also underwent apoptosis at a lower TMZ concentration. Lastly, exposure to a NF-κβ inhibitor significantly impaired TMZ resistance in GBM cells transfected with a *H19*-overexpressing plasmid.^32^

### LncRNA and Metabolism

Hypoxia is one of the key characteristics of GBM and glioma cells shift to aerobic glycolysis as a main source of ATP, a phenomenon referred to as the Warburg effect.^84^ As one of the chief glycolytic enzymes and upregulated by HIF-1α, HK2 is a compelling metabolic target because of its strong presence in gliomas and its role in maintaining high glycolytic rates. Elevated expression of HK2 is also associated with TMZ-resistant GBM tissues and poor survival in patients. Importantly, HK2 inhibition has been shown to restore oxidative glucose metabolism and decrease the overall tumorigenesis in GBM cell lines.^33,85^ Currently, HOX antisense intergenic RNA (*HOTAIR*) is the only lncRNA found to mediate its TMZ effects through HK2 in GBM.

#### HOTAIR.

— *HOTAIR* is highly expressed in GBM tissues and serum-derived extracellular vesicles, serving as a negative prognostic factor.^34,86^ Moreover, *HOTAIR* was found to be upregulated in TMZ-resistant GBM tissues and U87 and A172 GBM cells. A recent investigation described increased TMZ sensitivity in U87 and A172 GBM cells transfected with shRNA targeting *HOTAIR* (sh-*HOTAIR*). Zhang et al. treated sh-*HOTAIR* U87 and A172 cells with TMZ and analyzed the gene expressions. They found that the cells not only exhibited upregulated *miR-125* and decreased *HK2* levels, but also had increased apoptotic response to TMZ. Lastly, depletion of *HOTAIR* also enhanced the therapeutic effects of TMZ in vivo. Mouse xenograft models of sh-*HOTAIR* U87 cells were more sensitive to TMZ, leading to significantly smaller tumor and longer survival. Furthermore, the sh-*HOTAIR* U87 cells had suppressed HK2 and enhanced apoptotic protein levels. Collectively, these results indicate that *HOTAIR* regulates GBM TMZ response through the expression of *miR-125* and HK2.^33^

## Targeting LncRNA

Therapeutic regulation of lncRNAs represents an attractive approach for GBMs as they are highly tissue- and cell-type specific.^10^ To target non-coding RNAs, multiple approaches including siRNA, antisense oligonucleotides (ASOs), and the CRISPR/Cas9 system have been investigated.^87^

siRNAs are short double-stranded RNAs between 19 and 30 nucleotides. They induce gene silencing by recruiting the RNA-induced silencing complex to degrade the target gene posttranscriptionally.^88^ Although siRNAs can effectively target lncRNAs regardless of their intracellular locations, they are susceptible to nucleases and have lower bioavailability due to their large size and anionic charge. However, chemical modifications have improved their stability, specificity, and delivery.^88,89^ siRNA drugs have been approved for diseases such as adult hereditary amyloidogenic transthyretin.^90^

ASOs are single-stranded ASOs that are taken up freely by cells and form heteroduplexes with the complementary lncRNA. ASOs can inhibit or alter gene expression via steric hinderance, splicing alterations, or cleavage induction by endogenous RNaseH.^91^ ASOs have similarly undergone modifications of their backbone to improve their stability and delivery. Compared to siRNAs, ASOs are smaller, less immunogenic, and exhibit fewer off-target effects; the development of locked nucleic acids has also improved the potentcy significantly, though with increase hepatoxicity.^92,93^ Moreover, certain ASOs have demonstrated lower RNAse H cleavage and can only enter the central nervous system via intrathecal injections.^94^ Clinically, ASO-mediated therapies are currently used for treating diseases such as spinal muscular atrophy.^95^

The CRISPR/Cas9 system is composed of a single guide (sgRNA) and a Cas9 enzyme. The sgRNA guides the Cas9 nuclease to specific sites in the genome via complementary base pairing. The high efficiency and ease of programming makes CRISPR/Cas9 a robust tool to target lncRNAs.^96^ Furthermore, CRISPR/Cas9 can be utilized to target splice sites to produce intron retention or exon deletion, which can be used for genome-wide screening for essential lncRNAs.^97^ However, one major limitation is the risk of impacting adjacent genes, such as those that may overlap with the target lncRNA gene or those with DNA elements in the lncRNA locus that may regulate other genes.^98^ Unlike siRNAs and ASOs, CRISPR/Cas9 drugs have not yet been approved for treating diseases.

## Conclusion

TMZ resistance is a significant barrier to improving the dire prognosis of GBM. From the current aspects of recent research, lncRNAs are being increasingly recognized for their diverse roles in modulating various GBM pathologies. To the best of our knowledge, this review is the first to focus on and describe, in detail, the lncRNAs specifically involved in TMZ response in GBM. Furthermore, this review concurrently explores the mechanisms commonly associated with GBM chemo-resistance in the context of lncRNAs. We divided lncRNA-mediated TMZ resistance into three large categories: (1) regulation of the DDR, (2) maintenance of GSC identity, and (3) association with tumor hypoxia. Naturally, there are other existing mechanisms that do not fit in the above categories included in Table 1.

LncRNAs undoubtedly command a much more critical role in GBM pathogenesis than previously thought. The complex network between lncRNAs and TMZ resistance are only beginning to transpire and would require considerably more experiments to thoroughly understand and appreciate. Nevertheless, the highly-specific differential lncRNA expressions between normal and glioma cells, in conjunction with rapidly advancing sequence-based nucleic acid therapies emphasize lncRNAs as promising targets for novel GBM therapies.

## References

[1] Omuro A , DeAngelis LM. Glioblastoma and other malignant gliomas: a clinical review. JAMA. 2013;310(17):1842–1850. 24193082 10.1001/jama.2013.280319

[2] Davis ME . Glioblastoma: overview of disease and treatment. Clin J Oncol Nurs. 2016;20(5 suppl):S2–S8. 10.1188/16.CJON.S1.2-8 PMC5123811 27668386

[3] Lee SY . Temozolomide resistance in glioblastoma multiforme. Genes Dis. 2016;3(3):198–210. 30258889 10.1016/j.gendis.2016.04.007 PMC6150109

[4] Stupp R , Mason WP, van den Bent MJ, et al Radiotherapy plus concomitant and adjuvant temozolomide for glioblastoma. N Engl J Med. 2005;352(10):987–996. 15758009 10.1056/NEJMoa043330

[5] Hegi ME , Diserens AC, Gorlia T, et al MGMT gene silencing and benefit from temozolomide in glioblastoma. N Engl J Med. 2005;352(10):997–1003. 15758010 10.1056/NEJMoa043331

[6] Chen J , Li Y, Yu TS, et al A restricted cell population propagates glioblastoma growth after chemotherapy. Nature 2012;488(7412):522–526. 22854781 10.1038/nature11287 PMC3427400

[7] Liu L , Markowitz S, Gerson SL. Mismatch repair mutations override alkyltransferase in conferring resistance to temozolomide but not to 1,3-bis(2-chloroethyl)nitrosourea. Cancer Res. 1996;56(23):5375–5379. 8968088

[8] Lo Dico A , Martelli C, Diceglie C, Lucignani G, Ottobrini L. Hypoxia-inducible factor-1α activity as a switch for glioblastoma responsiveness to temozolomide. Front Oncol. 2018;8:249. 30013951 10.3389/fonc.2018.00249 PMC6036118

[9] Wu P , Zuo X, Deng H, et al Roles of long noncoding RNAs in brain development, functional diversification and neurodegenerative diseases. Brain Res Bull. 2013;97:69–80. 23756188 10.1016/j.brainresbull.2013.06.001

[10] Peng Z , Liu C, Wu M. New insights into long noncoding RNAs and their roles in glioma. Mol Cancer. 2018;17(1):61. 29458374 10.1186/s12943-018-0812-2 PMC5817731

[11] Katsushima K , Natsume A, Ohka F, et al Targeting the Notch-regulated non-coding RNA TUG1 for glioma treatment. Nat Commun. 2016;7:13616. 27922002 10.1038/ncomms13616 PMC5150648

[12] Hu GW , Wu L, Kuang W, et al Knockdown of linc-OIP5 inhibits proliferation and migration of glioma cells through down-regulation of YAP-NOTCH signaling pathway. Gene. 2017;610:24–31. 28189759 10.1016/j.gene.2017.02.006

[13] Sun SL , Shu YG, Tao MY. LncRNA CCAT2 promotes angiogenesis in glioma through activation of VEGFA signalling by sponging miR-424. Mol Cell Biochem. 2020;468(1–2):69–82. 32236863 10.1007/s11010-020-03712-y

[14] Ghafouri-Fard S , Agabalazadeh A, Abak A, et al Role of long non-coding RNAs in conferring resistance in tumors of the nervous system. Front Oncol. 2021;11:670917. 34178658 10.3389/fonc.2021.670917 PMC8219921

[15] Wu P , Cai J, Chen Q, et al Lnc-TALC promotes O^6^-methylguanine-DNA methyltransferase expression via regulating the c-Met pathway by competitively binding with miR-20b-3p. Nat Commun. 2019;10(1):2045. 31053733 10.1038/s41467-019-10025-2 PMC6499807

[16] Shangguan W , Lv X, Tian N. FoxD2-AS1 is a prognostic factor in glioma and promotes temozolomide resistance in a O^6^-methylguanine-DNA methyltransferase-dependent manner. Korean J Physiol Pharmacol. 2019;23(6):475–482. 31680769 10.4196/kjpp.2019.23.6.475 PMC6819902

[17] Gu N , Wang X, Di Z, et al Silencing lncRNA FOXD2-AS1 inhibits proliferation, migration, invasion and drug resistance of drug-resistant glioma cells and promotes their apoptosis via microRNA-98-5p/CPEB4 axis. Aging (Albany NY). 2019;11(22):10266–10283. 31770107 10.18632/aging.102455 PMC6914387

[18] Cheng M , Wang Q, Chen L, et al LncRNA UCA1/miR-182-5p/MGMT axis modulates glioma cell sensitivity to temozolomide through MGMT-related DNA damage pathways. Hum Pathol. 2022;123:59–73. 35219686 10.1016/j.humpath.2022.02.016

[19] Du P , Zhao H, Peng R, et al LncRNA-XIST interacts with miR-29c to modulate the chemoresistance of glioma cell to TMZ through DNA mismatch repair pathway. Biosci Rep. 2017;37(5):BSR20170696. 28831025 10.1042/BSR20170696 PMC5587918

[20] Zhang Z , Yin J, Lu C, et al Exosomal transfer of long non-coding RNA SBF2-AS1 enhances chemoresistance to temozolomide in glioblastoma. J Exp Clin Cancer Res. 2019;38(1):166. 30992025 10.1186/s13046-019-1139-6 PMC6469146

[21] Mazor G , Levin L, Picard D, et al The lncRNA TP73-AS1 is linked to aggressiveness in glioblastoma and promotes temozolomide resistance in glioblastoma cancer stem cells. Cell Death Dis. 2019;10(3):246. 30867410 10.1038/s41419-019-1477-5 PMC6416247

[22] Liu B , Zhou J, Wang C, et al LncRNA SOX2OT promotes temozolomide resistance by elevating SOX2 expression via ALKBH5-mediated epigenetic regulation in glioblastoma. Cell Death Dis. 2020;11(5):384. 32439916 10.1038/s41419-020-2540-y PMC7242335

[23] Bi CL , Liu JF, Zhang MY, et al LncRNA NEAT1 promotes malignant phenotypes and TMZ resistance in glioblastoma stem cells by regulating let-7g-5p/MAP3K1 axis. Biosci Rep. 2020;40(10). 10.1042/BSR20201111 PMC7601351 33057597

[24] Gao XY , Zang J, Zheng MH, et al Temozolomide treatment induces HMGB1 to promote the formation of glioma stem cells via the TLR2/NEAT1/Wnt pathway in glioblastoma. Front Cell Dev Biol. 2021;9:620883. 33614649 10.3389/fcell.2021.620883 PMC7891666

[25] Li B , Zhao H, Song J, Wang F, Chen M. LINC00174 down-regulation decreases chemoresistance to temozolomide in human glioma cells by regulating miR-138-5p/SOX9 axis. Hum Cell. 2020;33(1):159–174. 31713817 10.1007/s13577-019-00281-1

[26] Su YK , Lin JW, Shih JW, et al Targeting BC200/miR218-5p signaling axis for overcoming temozolomide resistance and suppressing glioma stemness. Cells. 2020;9(8):1859. 10.3390/cells9081859 PMC7463574 32784466

[27] Li H , Yuan X, Yan D, et al Long non-coding RNA MALAT1 decreases the sensitivity of resistant glioblastoma cell lines to temozolomide. Cell Physiol Biochem. 2017;42(3):1192–1201. 28668966 10.1159/000478917

[28] Cai T , Liu Y, Xiao J. Long noncoding RNA MALAT1 knockdown reverses chemoresistance to temozolomide via promoting microRNA-101 in glioblastoma. Cancer Med. 2018;7(4):1404–1415. 29479863 10.1002/cam4.1384 PMC5911628

[29] Chen W , Xu XK, Li JL, et al MALAT1 is a prognostic factor in glioblastoma multiforme and induces chemoresistance to temozolomide through suppressing miR-203 and promoting thymidylate synthase expression. Oncotarget. 2017;8(14):22783–22799. 28187000 10.18632/oncotarget.15199 PMC5410262

[30] Liu Y , Xu N, Liu B, et al Long noncoding RNA RP11-838N2.4 enhances the cytotoxic effects of temozolomide by inhibiting the functions of miR-10a in glioblastoma cell lines. Oncotarget. 2016;7(28):43835–43851. 27270310 10.18632/oncotarget.9699 PMC5190063

[31] Jia L , Tian Y, Chen Y, Zhang G. The silencing of LncRNA-H19 decreases chemoresistance of human glioma cells to temozolomide by suppressing epithelial-mesenchymal transition via the Wnt/β-Catenin pathway. Onco Targets Ther. 2018;11:313–321. 29391808 10.2147/OTT.S154339 PMC5769571

[32] Duan S , Li M, Wang Z, Wang L, Liu Y. H19 induced by oxidative stress confers temozolomide resistance in human glioma cells via activating NF-κB signaling. Onco Targets Ther. 2018;11:6395–6404. 30323617 10.2147/OTT.S173244 PMC6174297

[33] Zhang J , Chen G, Gao Y, Liang H. HOTAIR/miR-125 axis-mediated Hexokinase 2 expression promotes chemoresistance in human glioblastoma. J Cell Mol Med. 2020;24(10):5707–5717. 32279420 10.1111/jcmm.15233 PMC7214183

[34] Wang X , Yu X, Xu H, et al Serum-derived extracellular vesicles facilitate temozolomide resistance in glioblastoma through a HOTAIR-dependent mechanism. Cell Death Dis. 2022;13(4):344. 35418162 10.1038/s41419-022-04699-8 PMC9008004

[35] Xu N , Liu B, Lian C, et al Long noncoding RNA AC003092.1 promotes temozolomide chemosensitivity through miR-195/TFPI-2 signaling modulation in glioblastoma. Cell Death Dis. 2018;9(12):1139. 30442884 10.1038/s41419-018-1183-8 PMC6237774

[36] Liao Y , Shen L, Zhao H, et al LncRNA CASC2 interacts with miR-181a to modulate glioma growth and resistance to TMZ through PTEN pathway. J Cell Biochem. 2017;118(7):1889–1899. 28121023 10.1002/jcb.25910

[37] Shang C , Tang W, Pan C, Hu X, Hong Y. Long non-coding RNA TUSC7 inhibits temozolomide resistance by targeting miR-10a in glioblastoma. Cancer Chemother Pharmacol. 2018;81(4):671–678. 29397407 10.1007/s00280-018-3522-y

[38] Yan Y , Xu Z, Chen X, et al Novel function of lncRNA ADAMTS9-AS2 in promoting temozolomide resistance in glioblastoma via upregulating the FUS/MDM2 ubiquitination axis. Front Cell Dev Biol. 2019;7:217. 31632968 10.3389/fcell.2019.00217 PMC6783494

[39] Roos W , Baumgartner M, Kaina B. Apoptosis triggered by DNA damage *O*^6^-methylguanine in human lymphocytes requires DNA replication and is mediated by p53 and Fas/CD95/Apo-1. Oncogene. 2004;23(2):359–367. 14724564 10.1038/sj.onc.1207080

[40] Tang JB , Svilar D, Trivedi RN, et al *N*-methylpurine DNA glycosylase and DNA polymerase beta modulate BER inhibitor potentiation of glioma cells to temozolomide. Neuro Oncol. 2011;13(5):471–486. 21377995 10.1093/neuonc/nor011 PMC3093332

[41] Li J , Ji X, Wang H. Targeting long noncoding RNA HMMR-AS1 suppresses and radiosensitizes glioblastoma. Neoplasia 2018;20(5):456–466. 29574252 10.1016/j.neo.2018.02.010 PMC5915996

[42] Wiewrodt D , Nagel G, Dreimüller N, et al MGMT in primary and recurrent human glioblastomas after radiation and chemotherapy and comparison with p53 status and clinical outcome. Int J Cancer. 2008;122(6):1391–1399. 18000822 10.1002/ijc.23219

[43] Weller M , Tabatabai G, Kästner B, et al MGMT promoter methylation is a strong prognostic biomarker for benefit from dose-intensified temozolomide rechallenge in progressive glioblastoma: the DIRECTOR trial. Clin Cancer Res. 2015;21(9):2057–2064. 25655102 10.1158/1078-0432.CCR-14-2737

[44] Quinn JA , Desjardins A, Weingart J, et al Phase I trial of temozolomide plus *O*^6^-benzylguanine for patients with recurrent or progressive malignant glioma. J Clin Oncol. 2005;23(28):7178–7187. 16192602 10.1200/JCO.2005.06.502

[45] Wang J , Li B, Wang C, et al Long noncoding RNA FOXD2-AS1 promotes glioma cell cycle progression and proliferation through the FOXD2-AS1/miR-31/CDK1 pathway. J Cell Biochem. 2019;120(12):19784–19795. 31347720 10.1002/jcb.29284

[46] Yip S , Miao J, Cahill DP, et al MSH6 mutations arise in glioblastomas during temozolomide therapy and mediate temozolomide resistance. Clin Cancer Res. 2009;15(14):4622–4629. 19584161 10.1158/1078-0432.CCR-08-3012 PMC2737355

[47] Stritzelberger J , Distel L, Buslei R, Fietkau R, Putz F. Acquired temozolomide resistance in human glioblastoma cell line U251 is caused by mismatch repair deficiency and can be overcome by lomustine. Clin Transl Oncol. 2018;20(4):508–516. 28825189 10.1007/s12094-017-1743-x

[48] Naumann SC , Roos WP, Jöst E, et al Temozolomide- and fotemustine-induced apoptosis in human malignant melanoma cells: response related to MGMT, MMR, DSBs, and p53. Br J Cancer. 2009;100(2):322–333. 19127257 10.1038/sj.bjc.6604856 PMC2634706

[49] Gil Del Alcazar CR , Todorova PK, Habib AA, Mukherjee B, Burma S. Augmented HR repair mediates acquired temozolomide resistance in glioblastoma. Mol Cancer Res. 2016;14(10):928–940. 27358111 10.1158/1541-7786.MCR-16-0125 PMC5065752

[50] Zhu J , Kong F, Xing L, Jin Z, Li Z. Prognostic and clinicopathological value of long noncoding RNA XIST in cancer. Clin Chim Acta. 2018;479:43–47. 29307668 10.1016/j.cca.2018.01.005

[51] Cheng Z , Li Z, Ma K, et al Long non-coding RNA XIST promotes glioma tumorigenicity and angiogenesis by acting as a molecular sponge of miR-429. J Cancer. 2017;8(19):4106–4116. 29187887 10.7150/jca.21024 PMC5706014

[52] Fu J , Liu ZG, Liu XM, et al Glioblastoma stem cells resistant to temozolomide-induced autophagy. Chin Med J (Engl). 2009;122(11):1255–1259. 19567133

[53] Rossi DJ , Bryder D, Seita J, et al Deficiencies in DNA damage repair limit the function of haematopoietic stem cells with age. Nature 2007;447(7145):725–729. 17554309 10.1038/nature05862

[54] Liu G , Yuan X, Zeng Z, et al Analysis of gene expression and chemoresistance of CD133^+^ cancer stem cells in glioblastoma. Mol Cancer. 2006;5:67. 17140455 10.1186/1476-4598-5-67 PMC1697823

[55] Zhang X , Kiang KM, Zhang GP, Leung GK. Long non-coding RNAs dysregulation and function in glioblastoma stem cells. Noncoding RNA 2015;1(1):69–86. 29861416 10.3390/ncrna1010069 PMC5932540

[56] Brodie S , Lee HK, Jiang W, et al The novel long non-coding RNA TALNEC2, regulates tumor cell growth and the stemness and radiation response of glioma stem cells. Oncotarget. 2017;8(19):31785–31801. 28423669 10.18632/oncotarget.15991 PMC5458248

[57] Tang T , Wang LX, Yang ML, Zhang RM. lncRNA TPTEP1 inhibits stemness and radioresistance of glioma through miR-106a-5p-mediated P38 MAPK signaling. Mol Med Rep. 2020;22(6):4857–4867. 33173989 10.3892/mmr.2020.11542 PMC7646932

[58] Li H , Li H, Hao Y, et al Differential long non-coding RNA and mRNA expression in differentiated human glioblastoma stem cells. Mol Med Rep. 2016;14(3):2067–2076. 27432080 10.3892/mmr.2016.5505

[59] Schäfer A , Teufel J, Ringel F, et al Aldehyde dehydrogenase 1A1--a new mediator of resistance to temozolomide in glioblastoma. Neuro Oncol. 2012;14(12):1452–1464. 23132408 10.1093/neuonc/nos270 PMC3499020

[60] Wang Y , Wu N, Luo X, et al SOX2OT, a novel tumor-related long non-coding RNA. Biomed Pharmacother. 2020;123:109725. 31865145 10.1016/j.biopha.2019.109725

[61] Shahryari A , Jazi MS, Samaei NM, Mowla SJ. Long non-coding RNA SOX2OT: expression signature, splicing patterns, and emerging roles in pluripotency and tumorigenesis. Front Genet. 2015;6:196. 26136768 10.3389/fgene.2015.00196 PMC4469893

[62] Suvà ML , Rheinbay E, Gillespie SM, et al Reconstructing and reprogramming the tumor-propagating potential of glioblastoma stem-like cells. Cell. 2014;157(3):580–594. 24726434 10.1016/j.cell.2014.02.030 PMC4004670

[63] Garros-Regulez L , Garcia I, Carrasco-Garcia E, et al Targeting SOX2 as a therapeutic strategy in glioblastoma. Front Oncol. 2016;6:222. 27822457 10.3389/fonc.2016.00222 PMC5075570

[64] Bulstrode H , Johnstone E, Marques-Torrejon MA, et al Elevated FOXG1 and SOX2 in glioblastoma enforces neural stem cell identity through transcriptional control of cell cycle and epigenetic regulators. Genes Dev. 2017;31(8):757–773. 28465359 10.1101/gad.293027.116 PMC5435889

[65] Luo W , Yan D, Song Z, et al miR-126-3p sensitizes glioblastoma cells to temozolomide by inactivating Wnt/β-catenin signaling via targeting SOX2. Life Sci. 2019;226:98–106. 30980849 10.1016/j.lfs.2019.04.023

[66] Zhang Y , Lun L, Li H, et al The value of lncRNA NEAT1 as a prognostic factor for survival of cancer outcome: a meta-analysis. Sci Rep. 2017;7(1):13080. 29026116 10.1038/s41598-017-10001-0 PMC5638961

[67] Zhang XH , Qian Y, Li Z, Zhang NN, Xie YJ. Let-7g-5p inhibits epithelial-mesenchymal transition consistent with reduction of glioma stem cell phenotypes by targeting VSIG4 in glioblastoma. Oncol Rep. 2016;36(5):2967–2975. 27634309 10.3892/or.2016.5098

[68] Wang J , Zuo J, Wahafu A, et al Combined elevation of TRIB2 and MAP3K1 indicates poor prognosis and chemoresistance to temozolomide in glioblastoma. CNS Neurosci Ther. 2020;26(3):297–308. 31318172 10.1111/cns.13197 PMC7053231

[69] Wu H , Wang C, Liu Y, et al miR-138-5p suppresses glioblastoma cell viability and leads to cell cycle arrest by targeting cyclin D3. Oncol Lett. 2020;20(5):264. 32989398 10.3892/ol.2020.12127 PMC7517571

[70] Wang Z , Xu X, Liu N, et al SOX9-PDK1 axis is essential for glioma stem cell self-renewal and temozolomide resistance. Oncotarget 2018;9(1):192–204. 29416606 10.18632/oncotarget.22773 PMC5787456

[71] Zhan P , Wang J, Lv XJ, et al Prognostic value of vascular endothelial growth factor expression in patients with lung cancer: a systematic review with meta-analysis. J Thorac Oncol. 2009;4(9):1094–1103. 19687765 10.1097/JTO.0b013e3181a97e31

[72] Galmarini FC , Galmarini CM, Sarchi MI, Abulafia J, Galmarini D. Heterogeneous distribution of tumor blood supply affects the response to chemotherapy in patients with head and neck cancer. Microcirculation. 2000;7(6 Pt 1):405–410. 11142337

[73] Ping YF , Yao XH, Jiang JY, et al The chemokine CXCL12 and its receptor CXCR4 promote glioma stem cell-mediated VEGF production and tumour angiogenesis via PI3K/AKT signalling. J Pathol. 2011;224(3):344–354. 21618540 10.1002/path.2908

[74] Zhang T , Wang F, Liao Y, Yuan L, Zhang B. LncRNA AWPPH promotes the invasion and migration of glioma cells through the upregulation of HIF1α. Oncol Lett. 2019;18(6):6781–6786. 31807187 10.3892/ol.2019.11018 PMC6876321

[75] Joseph JV , Conroy S, Pavlov K, et al Hypoxia enhances migration and invasion in glioblastoma by promoting a mesenchymal shift mediated by the HIF1α-ZEB1 axis. Cancer Lett. 2015;359(1):107–116. 25592037 10.1016/j.canlet.2015.01.010

[76] Eastham AM , Spencer H, Soncin F, et al Epithelial-mesenchymal transition events during human embryonic stem cell differentiation. Cancer Res. 2007;67(23):11254–11262. 18056451 10.1158/0008-5472.CAN-07-2253

[77] O’Brien SJ , Bishop C, Hallion J, et al Long non-coding RNA (lncRNA) and epithelial-mesenchymal transition (EMT) in colorectal cancer: a systematic review. Cancer Biol Ther. 2020;21(9):769–781. 32730165 10.1080/15384047.2020.1794239 PMC7515495

[78] Gugnoni M , Ciarrocchi A. Long noncoding RNA and epithelial mesenchymal transition in cancer. Int J Mol Sci. 2019;20(8):1924. 10.3390/ijms20081924 PMC6515529 31003545

[79] Voce DJ , Bernal GM, Wu L, et al Temozolomide treatment induces lncRNA MALAT1 in an NF-κB and p53 codependent manner in glioblastoma. Cancer Res. 2019;79(10):2536–2548. 30940658 10.1158/0008-5472.CAN-18-2170 PMC6522287

[80] Siebzehnrubl FA , Silver DJ, Tugertimur B, et al The ZEB1 pathway links glioblastoma initiation, invasion and chemoresistance. EMBO Mol Med. 2013;5(8):1196–1212. 23818228 10.1002/emmm.201302827 PMC3944461

[81] Yan Y , Wang Q, Yan XL, et al miR-10a controls glioma migration and invasion through regulating epithelial-mesenchymal transition via EphA8. FEBS Lett. 2015;589(6):756–765. 25683004 10.1016/j.febslet.2015.02.005

[82] Hu Q , Yin J, Zeng A, et al H19 functions as a competing endogenous RNA to regulate EMT by sponging miR-130a-3p in glioma. Cell Physiol Biochem. 2018;50(1):233–245. 30282068 10.1159/000494002

[83] Yamini B . NF-κB, mesenchymal differentiation and glioblastoma. Cells. 2018;7(9):125. 10.3390/cells7090125 PMC6162779 30200302

[84] Warburg O . On respiratory impairment in cancer cells. Science. 1956;124(3215):269–270. 13351639

[85] Wolf A , Agnihotri S, Micallef J, et al Hexokinase 2 is a key mediator of aerobic glycolysis and promotes tumor growth in human glioblastoma multiforme. J Exp Med. 2011;208(2):313–326. 21242296 10.1084/jem.20101470 PMC3039857

[86] Zhou X , Ren Y, Zhang J, et al HOTAIR is a therapeutic target in glioblastoma. Oncotarget. 2015;6(10):8353–8365. 25823657 10.18632/oncotarget.3229 PMC4480757

[87] Chen Y , Li Z, Chen X, Zhang S. Long non-coding RNAs: from disease code to drug role. Acta Pharm Sin B. 2021;11(2):340–354. 33643816 10.1016/j.apsb.2020.10.001 PMC7893121

[88] Robb GB , Brown KM, Khurana J, Rana TM. Specific and potent RNAi in the nucleus of human cells. Nat Struct Mol Biol. 2005;12(2):133–137. 15643423 10.1038/nsmb886

[89] Lubini P , Zürcher W, Egli M. Stabilizing effects of the RNA 2′-substituent: crystal structure of an oligodeoxynucleotide duplex containing 2′-O-methylated adenosines. Chem Biol. 1994;1(1):39–45. 9383369 10.1016/1074-5521(94)90039-6

[90] Adams D , Gonzalez-Duarte A, O’Riordan WD, et al Patisiran, an RNAi therapeutic, for hereditary transthyretin amyloidosis. N Engl J Med. 2018;379(1):11–21. 29972753 10.1056/NEJMoa1716153

[91] Walder RY , Walder JA. Role of RNase H in hybrid-arrested translation by antisense oligonucleotides. Proc Natl Acad Sci USA. 1988;85(14):5011–5015. 2839827 10.1073/pnas.85.14.5011 PMC281677

[92] Swayze EE , Siwkowski AM, Wancewicz EV, et al Antisense oligonucleotides containing locked nucleic acid improve potency but cause significant hepatotoxicity in animals. Nucleic Acids Res. 2007;35(2):687–700. 17182632 10.1093/nar/gkl1071 PMC1802611

[93] Burel SA , Hart CE, Cauntay P, et al Hepatotoxicity of high affinity gapmer antisense oligonucleotides is mediated by RNase H1 dependent promiscuous reduction of very long pre-mRNA transcripts. Nucleic Acids Res. 2016;44(5):2093–2109. 26553810 10.1093/nar/gkv1210 PMC4797265

[94] Winkler J , Stessl M, Amartey J, Noe CR. Off-target effects related to the phosphorothioate modification of nucleic acids. ChemMedChem. 2010;5(8):1344–1352. 20544786 10.1002/cmdc.201000156

[95] Wood MJA , Talbot K, Bowerman M. Spinal muscular atrophy: antisense oligonucleotide therapy opens the door to an integrated therapeutic landscape. Hum Mol Genet. 2017;26(R02):R151–R159. 28977438 10.1093/hmg/ddx215

[96] Liu X , Homma A, Sayadi J, et al Sequence features associated with the cleavage efficiency of CRISPR/Cas9 system. Sci Rep. 2016;6:19675. 26813419 10.1038/srep19675 PMC4728555

[97] Liu Y , Cao Z, Wang Y, et al Genome-wide screening for functional long noncoding RNAs in human cells by Cas9 targeting of splice sites. Nat Biotechnol. 2018;36:1203–1210. 10.1038/nbt.4283 30395134

[98] Goyal A , Myacheva K, Groß M, et al Challenges of CRISPR/Cas9 applications for long non-coding RNA genes. Nucleic Acids Res. 2017;45(3):e12. 28180319 10.1093/nar/gkw883 PMC5388423

